# Photocatalytic Performance and Kinetic Studies of a Wood Surface Loaded with Bi_2_O_3_-Doped Silicon–Titanium Composite Film

**DOI:** 10.3390/polym15010025

**Published:** 2022-12-21

**Authors:** Zhigao Liu, Jinchi Xu, Si Cheng, Zhiyong Qin, Yunlin Fu

**Affiliations:** 1 College of Resources, Environment and Materials, Guangxi University, Nanning 530004, China; 2 College of Forestry, Guangxi University, Nanning 530004, China

**Keywords:** photocatalysis, wood surface, Bi_2_O_3_-doped titanium-silicon composite film, dynamics

## Abstract

In this paper, a surface self-cleaning wood was obtained by loading Bi_2_O_3_-doped silica–titanium composite film on the surface of wood by the sol–gel method. The effects of different Bi doping amounts on the structure and photocatalytic properties of the modified wood were investigated. The doping of Bi_2_O_3_ inhibited the growth of TiO_2_ crystals and the phase transition from anatase to rutile. In addition, Bi_2_O_3_ could improve the photocatalytic activity of the composite film by appropriately reducing the grain size of TiO_2_ and increasing the crystallinity of TiO_2_. Furthermore, doping with Bi_2_O_3_ shifted the absorption wavelength of the wood samples back into the visible range, indicating that the increase in Bi content favoured light absorption. The wood samples loaded with Bi_2_O_3_-doped Si–Ti composite membranes had the best photocatalytic activity and the highest reaction rate when n (Ti):n (Bi) = 1:0.015. Degradation rates of 96.0% and 94.0% could be achieved for rhodamine B and gaseous formaldehyde, respectively. It can be seen that wood samples loaded with Bi_2_O_3_-doped Si–Ti composite films on the surface exhibit excellent photocatalytic activity against both gaseous and liquid pollutants.

## 1. Introduction

Wood is widely used for construction and decoration because of its beautiful surface texture and unique environmental properties. The surface of wood is not only a major factor in determining the quality, value, and price of wood products, but also the most direct and sensitive part of human senses, which is important for the processing and use of wood [1]. However, the surface of wood is not only susceptible to liquid contamination, which affects its cleanliness and aesthetics, but also to microbial attack, mould, and decay, which shortens the life of the wood. Therefore, improving the properties of wood surfaces to enhance their efficacy and extend their service life has been a popular topic of research.

Formaldehyde, causing great harm to human beings, is the main gaseous pollutant produced in the process of interior decoration, artificial board making, and furniture finishing. Further, organic dye wastewater is one of the pollutants that have always existed in the printing and dyeing industry. Currently, the common treatment methods for formaldehyde and organic wastewater include adsorption [2,3], oxidation [4,5], biological treatment [6,7], and photocatalytic method [8,9], etc. As one of the best functional materials, TiO_2_ is also widely used for surface property improvement of wood due to its excellent chemical stability and non-toxicity. In water and air systems, TiO_2_ can be excited by UV light to generate photovoltaic electronics and holes and form highly chemically active radicals and reactive oxygen species on the surface. Therefore, it can react with most organic substances and decompose them into CO_2_ and H_2_O, resulting in the degradation of organic pollutants [10]. As early as 2002, Okawa et al. [11] prepared wood/TiO_2_ composites for the photocatalytic degradation of organic matter. Subsequently, Xia et al. [12] described the research progress of nano titanium dioxide photocatalytic modification from the reaction mechanism of nanotitanium dioxide photocatalytic degradation of formaldehyde and rationalized the feasibility of nanotitanium dioxide application in wood industry. Fu et al. [13] achieved photocatalytic degradation of the azo dye AO7 by SiO_2_/TiO_2_ modified materials prepared by a sol–gel method. Ag-TiO_2_ composite film-loaded wood capable of catalytic degradation of phenol under visible light irradiation was prepared by hydrothermal and silver mirror reactions by L. Gao [14] and L. Kun Gao [15], respectively. The former still had high photocatalytic activity after hydrophobic treatment, which provides a new possibility for the preparation of self-cleaning wood products.

In general, TiO_2_ has photocatalytic activity only under ultraviolet light, which is only a small fraction of sunlight. However, the wavelengths of light sources for indoor lighting are usually in the visible light range, which certainly limits the practical use and scope of TiO_2_-modified wood. Therefore, visible photocatalytic modification of TiO_2_ by elemental doping, noble metal deposition, semiconductor compounding, and ion doping is needed to broaden its photoreaction range and inhibit the compounding of photogenerated electrons-hole, resulting in the improvement of its photocatalytic activity. The forbidden band width of semiconductor Bi_2_O_3_ is 2.8 eV, which is narrower than that of TiO_2_, and therefore its absorption wavelength is longer. In addition, it has been shown [16,17,18,19,20] that Bi_2_O_3_ compounded with TiO_2_ can improve the photocatalytic activity of TiO_2_ and make it responsive in visible light. Therefore, a sol–gel method was used to load Bi_2_O_3_-doped silica–titanium composite films on the wood surface to improve the self-cleaning properties of the wood, and the effects of different Bi doping amounts on the surface morphology, chemical structure, crystal structure, and surface photocatalytic properties of the modified composite films were investigated.

## 2. Materials and Methods

### 2.1. Materials

Tsoongiodendron odorum wood was selected as test material, which was collected from Liangfengjiang National Forest Park, Nanning, Guangxi, China. The air-dried test material was processed into a 40 mm (L) × 40 mm (T) × 5 mm (R) sample size. The sample with a smooth surface and no defect was cleaned with distilled water and air-dried indoors (moisture content was about 15%) for later use.

### 2.2. Preparation of Composite Films

With the ratio of n (TBOT):n (EtOH):n (VETS):n (H_2_O):n (HNO_3_) being 1:5:0.2:1:0.5, the mixture of VETS and 1/3 EtOH were added to the mixture of TBOT and 1/3 EtOH under magnetic stirring, and then the mixture of 1/3 EtOH, H_2_O and HNO_3_ was added, and stirred vigorously for 10 min to obtain liquid A. BiCl_3_ was weighed at the molar ratio of n (Ti):n (Bi) (1:0, 0.005, 0.0075, 0.01, 0.015, 0.02) and dissolved in 0.2 mol of anhydrous ethanol and 0.1 mol of nitric acid mixture, respectively, and stirred vigorously for 10 min to obtain liquid B. Under vigorous stirring, liquid B was slowly dripped into liquid A and continued to be stirred for 1 h, then left at room temperature to form a sol. The sol was applied evenly to the surface of the wood and left for 1 h before a second application was made. After aging for 24 h, the wood was dried in a constant temperature oven at 100 °C for 6 h and then cooled to room temperature to obtain a series of wood samples with Bi^2^O^3^-doped silica–titanium composite films loaded on the surface, and the wood specimens loaded with different bismuth doping amounts (0.005, 0.0075, 0.01, 0.015, 0.02) of silica–titanium composite films were noted as BSTX, X = 0, 1, 2, 3, 4, 5.

### 2.3. Characterization of BSTX

#### 2.3.1. Morphology and Structural Characterization of Wood Surface

The surface morphology of Bi_2_O_3_-doped silicon titanium composite film on the wood surface was observed by stereomicroscope. A 300 kV emission transmission electron microscope was used to observe the lattice parameters of crystalline particles and judge their crystal structure. In this paper, the chemical structure of SiO_2_ and TiO_2_ in the composite film and its bonding mode with wood surface were analyzed by a NicoletiS 50 Fourier transform infrared spectrometer (Waltham, MA, USA). The crystallinity and crystal structure of SiO_2_ and TiO_2_ in the silicon titanium composite film were analyzed by a dX-2700A high-power (4 kW) polycrystalline X-ray diffractometer. The UV-visible diffuse reflectance spectra of the silica–titanium composite films doped with Bi_2_O_3_ on the wood surface were tested by UV-2501PC UV-visible spectrophotometer (Kyoto, Japan). BaSO_4_ was used as a reference, and the scanning range was 250–800 nm. The light emitted from the light source was processed into a sample of wood loaded with a bismuth-doped silica–titanium composite film and passed through an integrating sphere with an inner wall coated with BaSO4. The reflected light from the surface of the sample was collected and projected back to the receiver, where an electrical signal was generated and recorded on the recorder as a function of wavelength, resulting in a spectral curve. The band gaps of the samples were calculated using the Tauc equation (Equation (1)) based on the UV diffuse reflectance spectroscopy data [21].

(αhν)^1/2^ = A(hν − Eg) (1)

where h is Planck’s constant, ν is frequency of vibration, α is absorption coefficient, Eg is band gap, and A is proportional constant. 

#### 2.3.2. Characterization on Photocatalytic Performance

The photocatalytic performance of wood samples was evaluated by catalytic degradation of rhodamine B aqueous solution and gaseous formaldehyde under visible light.

**Figure 1:**
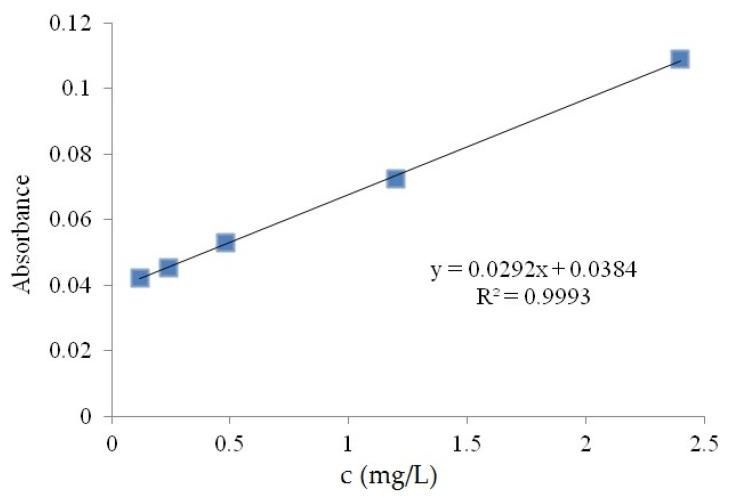
The standard curve of Rhodamine B aqueous solution.

Degradation of Rhodamine B: The standard curve of absorbance of rhodamine B aqueous solution changing with concentration was shown in Figure 1. A 500 W xenon lamp (λ > 420 nm) was selected as the light source, and a glass beaker was used as the reactor to immerse the wood sample with a Bi_2_O_3_-doped silica–titanium composite film into 30 mL of rhodamine B solution at a concentration of 10 mg/L. The samples were placed in the dark for 30 min and the concentration of Rhodamine B was determined as the initial concentration C_0_ after adsorption equilibrium was reached. The lamp was then turned on so that the surface of the wood with the composite film faced the light source vertically. The effective area of the wood sample was 40 mm × 40 mm, and the distance from the center of the light source was 40 cm. The reaction temperature was controlled at 20–30 °C. The illumination time was 180 min, and samples were taken every 30 min. The degradation rate of Rhodamine B was calculated by measuring the absorbance at the wavelength of 554 nm and converting it into concentration C. In the blank control group, wood samples without Bi_2_O_3_ doping were added. The decolorization rate D was calculated by the following equation.

D = [(C_0_ − C)/C_0_] × 100% (2)

**Figure 2:**
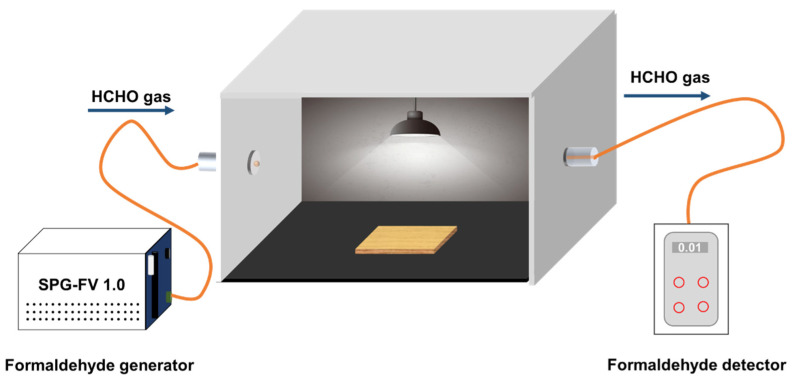
Reaction device for the photocatalytic degradation of formaldehyde.

Degradation of the gas formaldehyde: The formaldehyde standard solution with a mass fraction of 0.02 mg/mL was first configured. Then, 3 mL of the formaldehyde standard solution was converted into gas through the formaldehyde generator into the reaction device (Figure 2). The concentration of formaldehyde gas in the reaction chamber was measured with a formaldehyde detector after 30 min of adsorption in the dark as the initial concentration C_0_. The light was then turned on, keeping the wood surface with the composite film vertically facing the light source. The effective area of the wood sample was 40 mm × 40 mm, and the distance from the center of the light source was 40 cm. The reaction temperature was controlled at 20–30 °C and the illumination time was 180 min. The formaldehyde gas concentration in the reaction box was detected by a formaldehyde detector every 30 min. In blank control group, wood samples without Bi_2_O_3_ doping were added. The calculation of degradation rate D was the same as Formula (1).

#### 2.3.3. Photocatalytic Reaction Kinetic Model

The occurrence of a reaction, the direction of the reaction, and the concentration of substances involved in the reaction all depends on thermodynamics. Further, kinetics can be used to describe the reaction rate, the sequence of steps in the reaction, and the factors controlling the reaction rate. According to research, there are mainly two forms of photocatalytic reaction kinetics model: (1) Langmuir-Hinshelwood model (L-H model for short) [22], (2) Power law model [23].

In the L-H model, the reaction rate R is proportional to the reactant coverage θ on the membrane surface.

(3) $$R=k\theta =\frac{\mathrm{kKC}}{1+\mathrm{KC}}$$

where, k is the reaction rate constant, mol/min; K is the absorption coefficient of reactants on the membrane surface, m^3^/mol; C is the reactant concentration. When C is very small, KC < < 1, Formula (3) can be reduced to Equation (4) as shown below.

R = kKC(4)

Equation (4) is the kinetic equation of the first order reaction. First integrate this equation, when the initial reaction t = 0, C = C_0_ (C_0_ is the initial reaction concentration, C is the reaction concentration at time t), then the following equation can be obtained.

(5) $$-\mathrm{lnC}/C_{0}=k_{\mathrm{app}}t=\mathrm{kKt}$$

#### 2.3.4. Surface Wettability Test

The wettability of the solid surface is generally expressed by the contact angle θ of the liquid on the solid surface. If 0° < θ < 90°, the solid surface is hydrophilic; if 90° < θ < 180°, the solid surface is hydrophobic; and the larger θ is, the better the hydrophobicity of the solid surface is. In this paper, the static contact angle of distilled water on the wood surface was measured by a DSA100E Klux contact angle meter (Hamburg, Germany), the volume of water droplets was 5 μL, the contact angle of water droplets stayed on the wood surface for 10 s, the test temperature was 20 ± 1 °C, and the relative humidity was 65 ± 3%. Overall, 5 points were tested for each sample and 3 samples were tested for each treatment. Finally, the average value was taken as the contact angle on the surface of wood.

## 3. Results and Discussion

### 3.1. Analysis of Wood Surface Morphology

**Figure 3:**
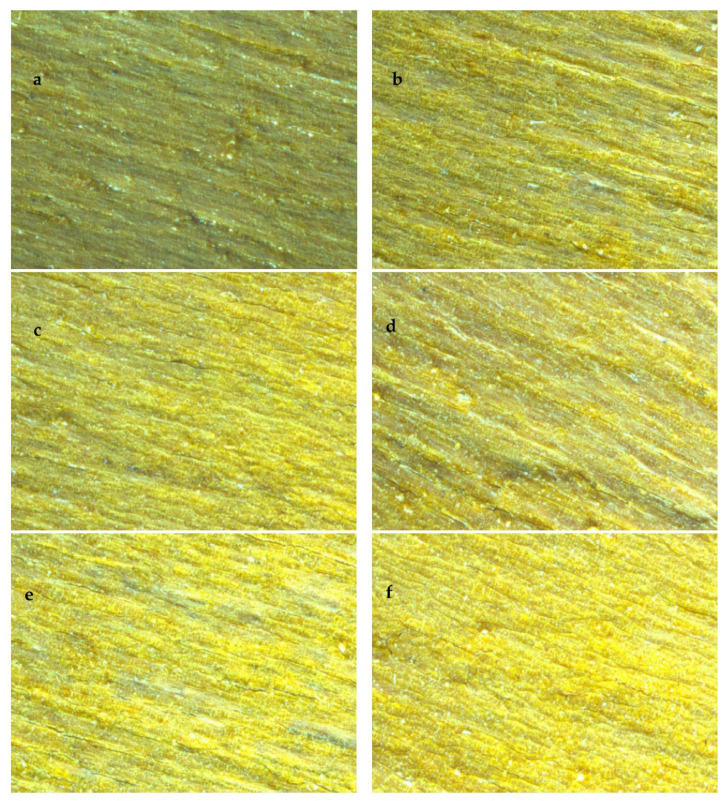
The stereoscopic microscope image of Si–Ti composite film prepared at different Bi_2_O_3_-doped amounts. ((**a**–**f**) are the stereoscopic microscope image of BST0, BST1, BST2, BST3, BST4, BST5).

**Figure 4:**
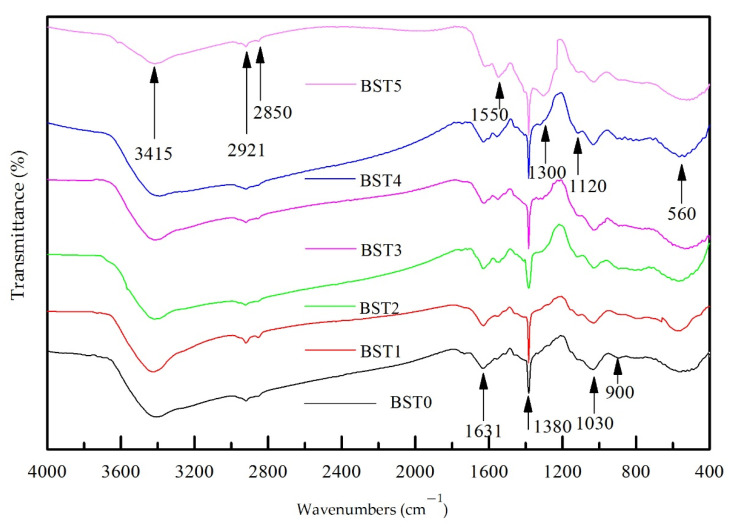
The FTIR spectrum of Si–Ti composite film prepared at different Bi-doped amounts.

According to the stereoscopic microscope view (magnification 28.5×) in Figure 3, a layer of yellow film was loaded on the treated wood surface, while some cracks appeared in the composite film after doping with Bi_2_O_3_. Combined with Figure 4, it may be due to the poor combination of Bi_2_O_3_ loaded on the wood surface with TiO_2_ and SiO_2_, and cracks due to different shrinkage, stress, and thermal expansion coefficients between particles and wood matrix during drying, which may be the reason for the decrease in contact angle [24].

### 3.2. FTIR Analysis

Figure 4 shows the FTIR patterns of samples with different Bi doping amounts. From the figure, it can be seen that doping of Bi_2_O_3_ makes some changes in the chemical groups of Si–Ti composite film on the wood surface. The broad and obtuse peak near 3415 cm^−1^ was the stretching vibration peak of -OH, partly from-OH in wood cellulose and partly from -OH produced by the hydrolysis of TBOT and VETS during the reaction. The peaks near 2921 cm^−1^ and 2850 cm^−1^ were the stretching vibration peaks of C-H in-CH_2_, which may come from the incomplete hydrolysis residue of wood cellulose, TBOT, and VETS, but the strength was very weak, so it was speculated that the hydrolysis reaction was sufficient and the residual -CH_2_ was lessened. The peak near 1631 cm^−1^ may be the stretching vibration peak of C=C, and its vibration intensity decreased with the increase in the content of Bi_2_O_3_. It was speculated that the doping of Bi_2_O_3_ would hinder the loading of -CH=CH_2_ on the wood surface, which may be the reason for the decrease in contact angle. The peak near 1550 cm^−1^ detected when n (Ti):n (Bi) ≥ 1:0.005 may be the stretching vibration peak of Bi-O, the peak near 1300 cm^−1^ detected by n (Ti):n (Bi) ≥ 1:0.075 cm^−1^ may be the stretching vibration peak of Bi-O [25], and the intensity of the vibration peak increased with the increase in the amount of Bi doping. It was speculated that Bi_2_O_3_ may be formed in the reaction. The sharp peak of 1380 cm^−1^ was the stretching vibration peak of -NO_3_. The peaks near 1120 cm^−1^ and 1030 cm^−1^ may be Si-O-Si asymmetric stretching vibration peaks. The peak near 900 cm^−1^ may be the stretching vibration of Ti-O-Si, but the peak strength was weak and not obvious, suggesting that the binding between TiO_2_ and SiO_2_ was weak. The peak near 560 cm^−1^ may be the stretching vibration peak of Ti-O-Ti. The appearance of Si-O-Si and Ti-O-Ti indicated that TiO_2_ and SiO_2_ were successfully supported on the wood surface.

### 3.3. Analysis of Crystallinity and Crystal Structure

**Figure 5:**
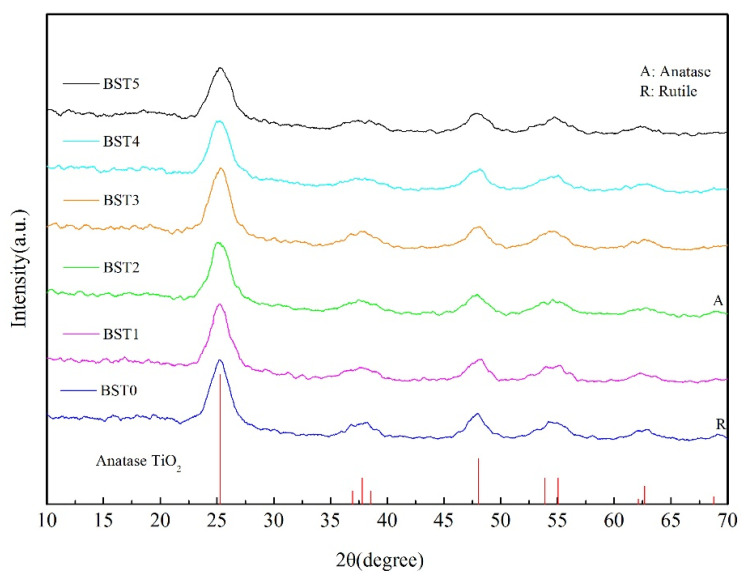
The XRD spectrum of Si–Ti composite film prepared at different Bi-doped amounts.

Figure 5 shows the XRD patterns of composite films on the surface of wood samples with different Bi doping levels. According to the figure analysis, when Bi was not doped, the composite film was mainly composed of anatase TiO_2_, but a small peak of rutile TiO_2_ appears at 2θ = 69.008°. After doping with Bi, the small peak of rutile TiO_2_ changed slightly, especially at the higher doping amount of bismuth where the peak basically disappeared, indicating that the composite film was composed of anatase TiO_2_ at this time. It can be concluded that the doping of Bi_2_O_3_ can inhibit the phase change of TiO_2_ from anatase to rutile in a certain extent. In addition, with the increase in Bi doping amount, the position of the TiO_2_ peak did not shift, and no new peak was generated, indicating that Bi and Ti did not generate new composite oxides. The average grain size of the BSTX composite film was calculated by the Scheler formula and the half-peak width of the anatase TiO_2_ (101) crystal plane. The results were shown in Table 1.

**Figure 6:**
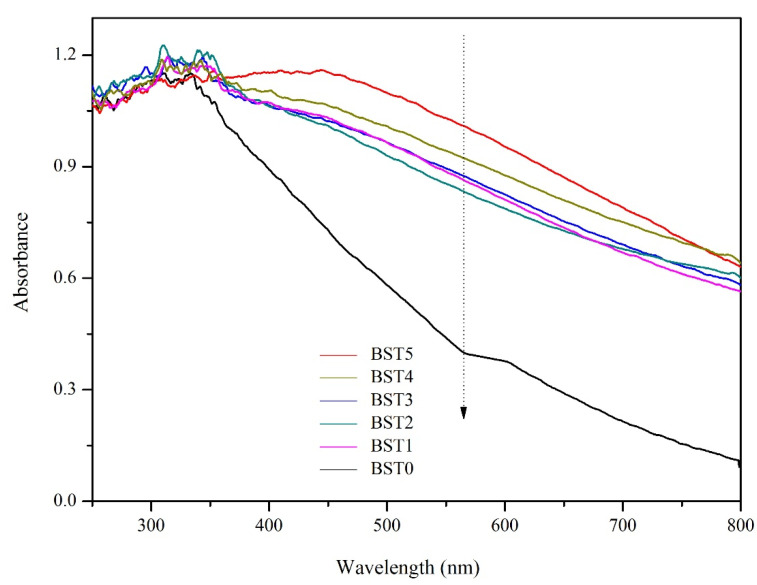
UV-Vis spectrum of Si–Ti composite film prepared at different Bi-doped amounts.

**Figure 7:**
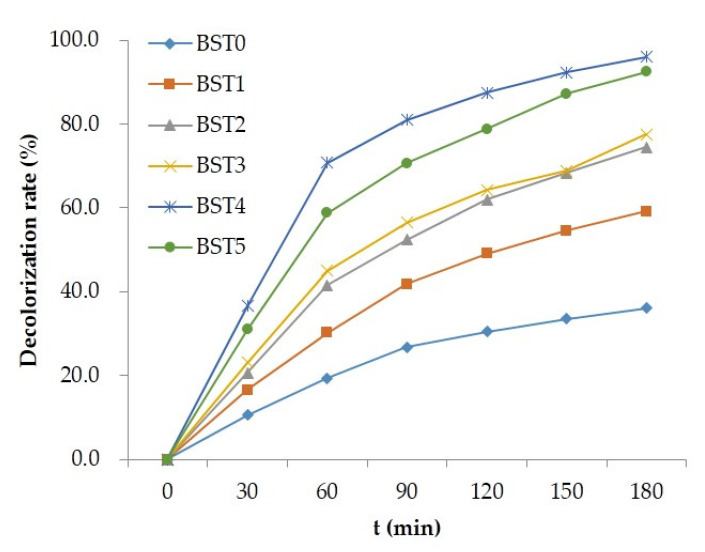
The decolorization rate of Rhodamine B from different Bi-doped wood samples.

The TiO_2_ microstructure such as energy band structure, degree of crystallinity, and crystal particle size may affect the photocatalytic activity of TiO_2_ [26]. It can be seen from Table 1 that with the increase in Bi doping amount, the average grain size of TiO_2_ decreased, indicating that Bi_2_O_3_ inhibits the growth of TiO_2_ crystals, which was consistent with previous studies [27]. In addition, the best photocatalytic efficiency of BST4 can be seen in Figure 6 and Figure 7, indicating that the reduction in crystal size within a certain range is beneficial for the increase in photocatalytic activity. With the increase in Bi doping amount, the crystallinity of TiO_2_ increased first and then decreased. When n (Ti):n (Bi) = 1:0.015, the crystallinity of TiO_2_ was the highest. The higher the crystallinity of the crystal, the fewer the internal defects and the corresponding reduction in the chance of electron-hole recombination; the high crystallinity would promote the rapid transfer of photo-generated electrons from the inside of the crystal to the surface, effectively inhibiting the photo-generated electron-hole recombination, thereby improving the quantum efficiency of photocatalysis and enhancing the photocatalytic activity [27].

**Figure 8:**
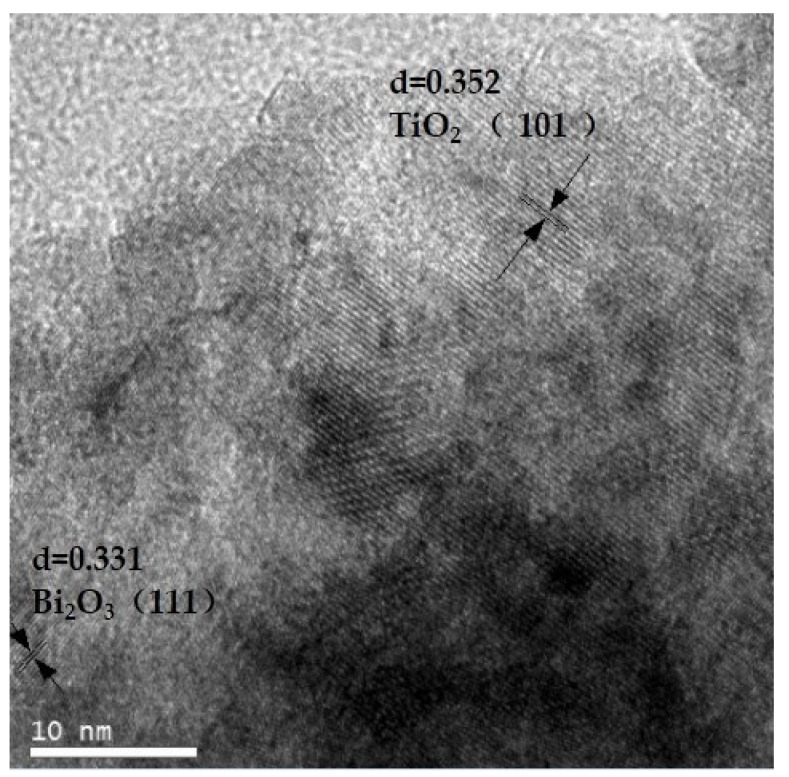
HRTEM image of Si–Ti composite film surface of the BSTS sample.

The HRTEM photo of the film layer on the surface of wood sample with n (Ti):n (Bi) = 1:0.015 is shown in Figure 8. As can be seen from the figure, clear lattice stripes can be seen in the BST3 sample. The spacing of these lattice stripes was 0.352 nm, corresponding to the 101 crystal plane of AnataseTiO_2_, which was consistent with the results of the XRD test. Although the stripes with lattice spacing of 0.331 nm correspond to the 111 crystal plane of α-Bi_2_O_3_, these lattice stripes were vague, which may be due to the small amount of Bi_2_O_3_ or the low crystallinity. 

### 3.4. UV-VIS Diffuse Reflectance Spectrum Analysis

Figure 6 shows the absorption spectrum calculated from the UV-vis diffuse reflection spectrum according to the Kubelka Munk (km) theory. As can be seen from Figure 6, the difference in absorption intensity of silicon–titanium composite film with different Bi doping amount was very small—between 250 nm–350 nm. The main absorption wavelength of wood loaded with silicon–titanium composite film was below 400 nm, while the wood coated with silicon–titanium composite film doped with Bi_2_O_3_ had strong light absorption capacity within the range of 400–800 nm. This indicated that the absorption wavelength of wood samples were red-shifted to the visible light range after Bi_2_O_3_ doping. As the Bi doping content increased, the light absorption ability of wood samples in the range of 400–800 nm was enhanced, indicating that the increase in Bi content was beneficial to light absorption, because the light response in the visible region was mainly from the photosensitization of Bi_2_O_3_ [28].

**Figure 9:**
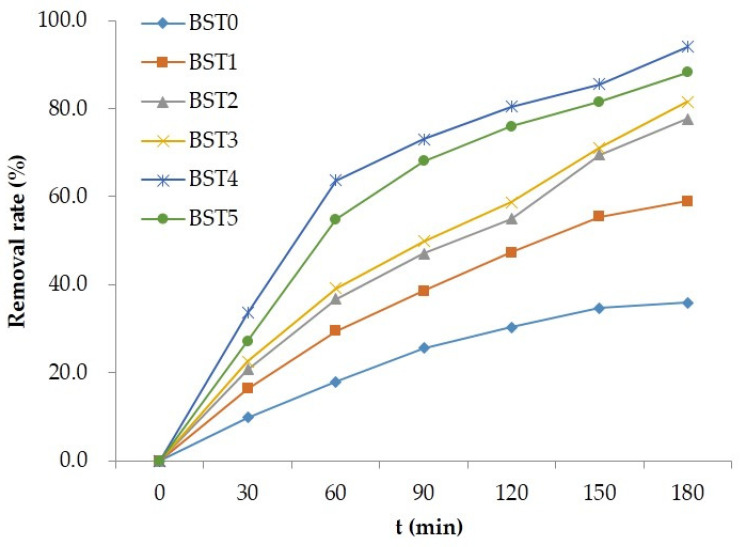
The removal rate of gas Formaldehyde in different Bi-doped wood samples.

As shown in the Table 2, the band gaps of the Si–Ti composite films without Bi doping and with Bi doping were 1.56, 1.36, 1.41, 1.34, 1.25, and 1.35 eV, respectively. It can be seen that the band gap of titanium dioxide in the Si–Ti composite film is reduced after doping with Bi, which makes the material absorb less light and shift toward the infrared direction, which will be beneficial to the improvement of the visible photocatalytic performance of the composite film. In addition, the decolorization and removal rates of BST4 for rhodamine B and formaldehyde (Figure 7 and Figure 9) were 96% and 94%, respectively, which indicated that the reduction in band gap had a certain promotion effect on the photocatalytic performance of the composite film.

### 3.5. Analysis of Photocatalytic Activity

The photocatalytic activity of Si–Ti composite film on the wood surface before and after Bi_2_O_3_ doping was evaluated by using liquid phase degradation of rhodamine B and gas phase degradation of formaldehyde under visible light as probe reaction. Figure 7 shows the degradation of Rhodamine B aqueous solution by wood samples loaded with different Bi_2_O_3_ doped silicon titanium composite films under visible light irradiation. It can be seen from the figure that under visible light irradiation, the photocatalytic activity of wood samples doped with Bi_2_O_3_ was obviously better than that of wood samples without Bi_2_O_3_, in which n (Ti):n (Bi) = 1:0.015 had the highest photocatalytic activity, and the decolorization rate of rhodamine B was 96.0%. Combining Figure 7 and Figure 8, it can be seen that the photocatalytic activity of wood samples loaded with Bi_2_O_3_-doped Si–Ti composite films depended on the amount of Bi doping, but excessive Bi content leaded to the agglomeration of Bi_2_O_3_, which became a photoelectron-hole recombination center and reduced the quantum efficiency, thus weakening the photocatalytic activity [29].

The wood samples loaded with Bi_2_O_3_-doped Si–Ti composite films showed excellent photocatalytic activity not only in the degradation of liquid phase dyes, but also in the removal of gaseous pollutant formaldehyde. As shown in Figure 8, similar to the result of degradation of rhodamine B, when n (Ti):n (Bi) = 1:0.015, the wood sample loaded with Bi_2_O_3_-doped silicon–titanium composite film had the best photocatalytic activity, with the removal rate of formaldehyde reaching 94.0%.

### 3.6. Analysis of Reaction Kinetics

**Figure 10:**
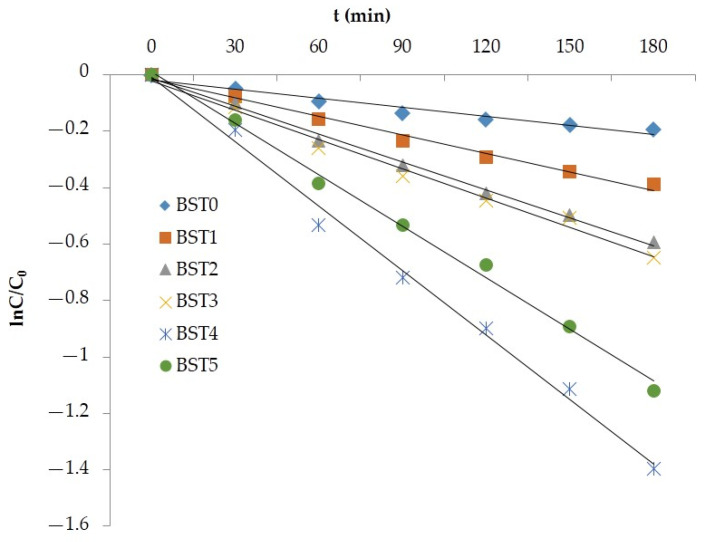
The reaction kinetics curves of degradation of RhB in different Bi-doped levels of wood samples.

**Figure 11:**
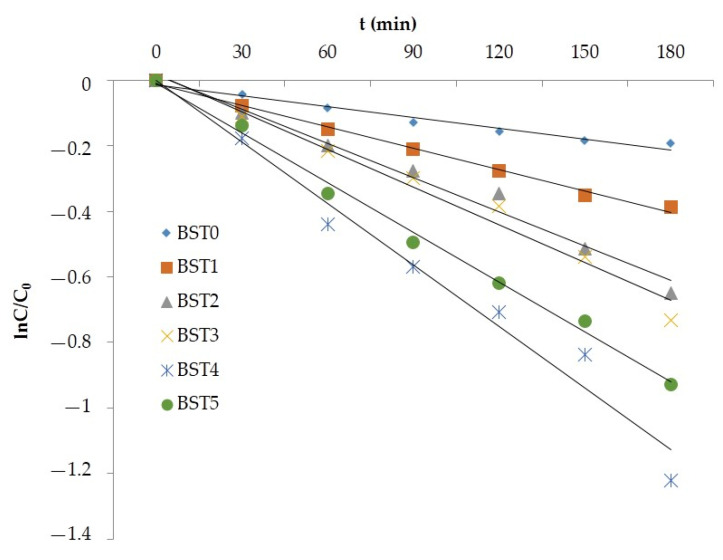
The reaction linetics curves of degradation of Gaseous formaldehyde in different Bi-doped levels of wood samples.

It can be seen from Equation (5) that if lnC/C_0_ was used to plot the reaction time T, a straight line should be obtained [30]. The reaction kinetics curves of photocatalytic degradation of rhodamine B and gaseous formaldehyde by Si–Ti composite film loaded on the wood surface with different Bi doping amount were obtained by plotting and fitting lnC/C_0_ to reaction time T, as shown in Figure 10 and Figure 11. It can be seen from the figure that the fitted lnC/C_0_~t was in a straight line, with the minimum R^2^ of 0.9587 and the maximum R^2^ of 0.9952, and that the fitting degree was high, indicating that the photocatalytic oxidation reaction of rhodamine B and formaldehyde on the surface of silicon titanium composite film with different Bi doping amount conforms to first-order reaction kinetics when rhodamine B and formaldehyde concentration were adsorbed on the surface. Therefore, in the photocatalytic degradation of rhodamine B and formaldehyde by silicon–titanium composite film with different Bi doping levels, the speed of the photocatalytic reaction on the surface of the composite film controlled the overall reaction rate rather than the adsorption process, indicating that the photocatalytic oxidation reaction rate of the composite film only depended on the speed of the photocatalytic reaction [31].

**Figure 12:**
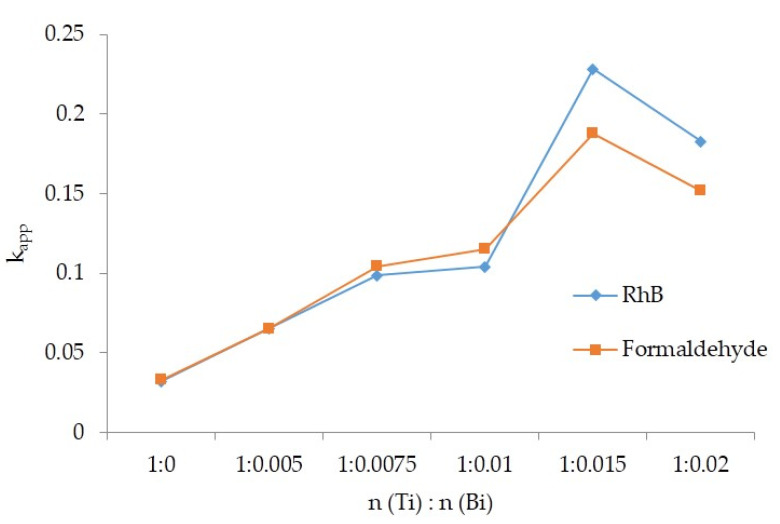
The rate constant of the apparent reaction of RhB and Gas Formaldehyde degraded by different Bi doping Si–Ti composite films.

Figure 12 shows the apparent reaction rate constant of degradation of rhodamine B and gaseous formaldehyde by Si–Ti composite films with different Bi doping amounts. As can be seen from the figure, with the increase in the amount of Bi doping, the change trend of the apparent reaction rate constant of the composite film to rhodamine B and gas formaldehyde was the same, which increased at first and then decreased. The apparent rate constants of the composite films for rhodamine B and gaseous formaldehyde were equal at n (Ti):n (Bi) of 1:0 and 1:0.005. When n (Ti):n (Bi) was 1:0.0075 and 1:0.01, the apparent reaction rate constant of the composite film for degrading gaseous formaldehyde was larger than that of rhodamine B. In contrast, the apparent reaction rate constants for the degradation of rhodamine B by the composite films were greater than that of formaldehyde when n (Ti):n (Bi) was 1:0.015 and 1:0.02. Moreover, the Si–Ti composite film doped with Bi^2^O^3^ at n (Ti):n (Bi) of 1:0.015 showed the highest degradation rates of 0.2283 and 0.1877 for rhodamine B and gaseous formaldehyde, respectively.

### 3.7. Analysis of Surface Wettability

**Figure 13:**
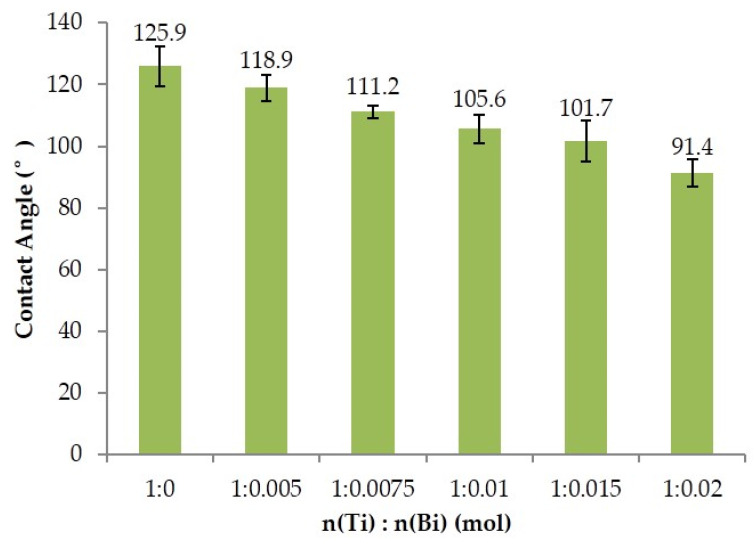
The contact angle of Si–Ti composite film prepared at different Bi-doped amounts.

The contact angles of silica–titanium composite films with different Bi doping amounts were shown in Figure 13. It can be seen from the figure that the contact angle of the wood specimens showed a decreasing trend with the increase in Bi doping amount; when the doping amount increased from 0 to 2%, the contact angle decreased from 125.9° to 91.4°, indicating that the increase in Bi_2_O_3_ would reduce the hydrophobicity of the silica–titanium composite film on the wood surface. Combined with the surface morphology and FTIR results, it was speculated that it may be due to the fact that the Bi_2_O_3_ loading on the surface of the wood increased with the increase in Bi doping. Alternatively, Bi_2_O_3_ did not bond well with TiO_2_ and SiO_2_, and cracks occurred during the drying process due to different drying stresses between the particles as well as between the wood [24]. Furthermore, the doping of Bi_2_O_3_ may hinder the loading of -CH=CH_2_ on the wood surface and thus the hydrophobic group decreases, leading to a decrease in hydrophobicity.

## 4. Conclusions

Wood samples loaded with Bi_2_O_3_-doped Si–Ti composite films were prepared by a sol–gel method in this paper. It can be inferred from the appearance of the Bi-O stretching vibration peaks that the wood surface was successfully loaded with Bi_2_O_3_, and the doping of Bi_2_O_3_ affected the loading of -CH=CH_2_ on the wood surface, which may be the reason for the decrease in contact angle. The doping of Bi_2_O_3_ inhibited the growth of TiO_2_ crystals and the phase transition from anatase to rutile. In addition, Bi_2_O_3_ reduced photoelectron-hole recombination by reducing the grain size of TiO_2_ and increasing the crystallinity of TiO_2_, which improved the photocatalytic activity. In addition, the reduction in TiO_2_ band gap after doping with Bi_2_O_3_ shifted the absorption wavelength of wood samples to the visible range, indicating that the increase in Bi content was beneficial to light absorption. The wood samples loaded with Bi_2_O_3_-doped silica–titanium composite films had the best photocatalytic activity and the highest reaction rate when n (Ti):n (Bi) = 1:0.015. The degradation rates of Rhodamine B and gaseous formaldehyde could reach 96.0% and 94.0%, respectively. The contact angle test showed that the contact angle of the wood samples decreased with increasing Bi content after doping with Bi_2_O_3_, which might be due to the reduction in cracks and hydrophobic groups on the surface of the composite film.

## Figures and Tables

**Table 1:** The average crystal grain size of Bi-Ti/Si-X composite film.

	ST0	BST1	BST2	BST3	BST4	BST5
**Grain size/nm**	93.49	87.65	82.27	76.94	71.77	56.19
**Crystallinity/%**	56.54	59.55	60.77	61.34	70.82	65.28

**Table 2:** The band gaps of Bi-Ti/Si-X composite films.

	ST0	BST1	BST2	BST3	BST4	BST5
**Eg (eV)**	1.56	1.36	1.41	1.34	1.25	1.35

## Data Availability

Not applicable.
